# Theoretical Assessment of Indistinguishable Peptides in Mass Spectrometry-Based Proteomics

**DOI:** 10.1021/acs.analchem.4c02803

**Published:** 2024-09-25

**Authors:** Zahra Elhamraoui, Eva Borràs, Mathias Wilhelm, Eduard Sabidó

**Affiliations:** † Centre for Genomic Regulation (CRG), The Barcelona Institute of Science and Technology (BIST), Dr. Aiguader 88, Barcelona 08003, Spain; ‡ Universitat Pompeu Fabra (UPF), Dr. Aiguader 88, Barcelona 08003, Spain; § Computational Mass Spectrometry, Technical University of Munich, D-85354 Freising, Germany; # Munich Data Science Institute (MDSI), Technical University of Munich, D-85748 Garching, Germany

## Abstract

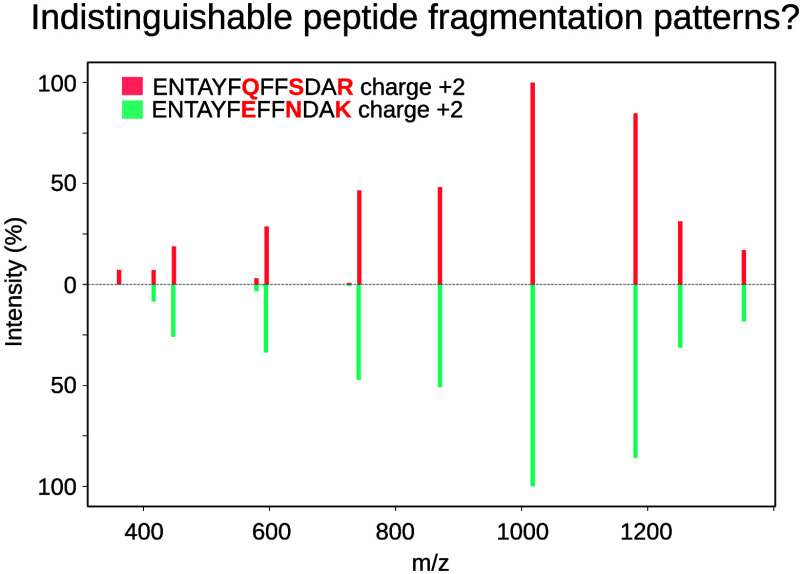

Mass-spectrometry-based proteomics has advanced with the integration of experimental and predicted spectral libraries, which have significantly improved peptide identification in complex search spaces. However, challenges persist in distinguishing some peptides with close retention times and nearly identical fragmentation patterns. In this study, we conducted a theoretical assessment to quantify the prevalence of indistinguishable peptides within the human canonical proteome and immunopeptidome using state-of-the-art retention time and spectrum prediction models. By quantifying the proportion of peptides posing challenges to unequivocal identification, we set the theoretical nonaccessible portion within a given proteome, and underscore the effectiveness of contemporary analytical methodologies in resolving the complexity of the human proteome and immunopeptidome via mass spectrometry.

Mass-spectrometry-based proteomics mostly relies on the fragmentation of peptides for deducing their amino acid sequence.^1,2^ The use of experimental spectral libraries and, more recently, the new machine learning models capable of predicting peptide properties, have introduced new dimensionalities (e.g., retention time and fragment relative intensities) that have significantly improved the confidence in peptide detection and identification in complex search spaces.^3−7^

Despite the increase of available information to interpret and assign peptide fragmentation spectra to given sequences, there are still peptides that show similar analytical values in experimental data, e.g., close retention time and nearly identical fragmentation patterns.^6^ For instance, when analyzing experimental proteomics results from commercial HeLa tryptic digests acquired in DIA mode, several peptide assignments can be found showing similar retention time and fragmentation patterns (see Figure 1A, as well as the Supporting Information).^8^ These cases include obvious instances, such as sequences featuring an Ile/Leu substitution, but also peptides bearing small amino acid permutations, or even unrelated sequence changes (Figure S1). The similarity in retention time and fragmentation relative intensities of certain peptide sequences make them virtually indistinguishable with the current available data analysis tools. This challenges accurate peptide identification, regardless of the mass spectrometry data acquisition strategy (i.e., DDA, DIA, PRM, etc.), and independently of the false discovery rate estimation.

**Figure 1:**
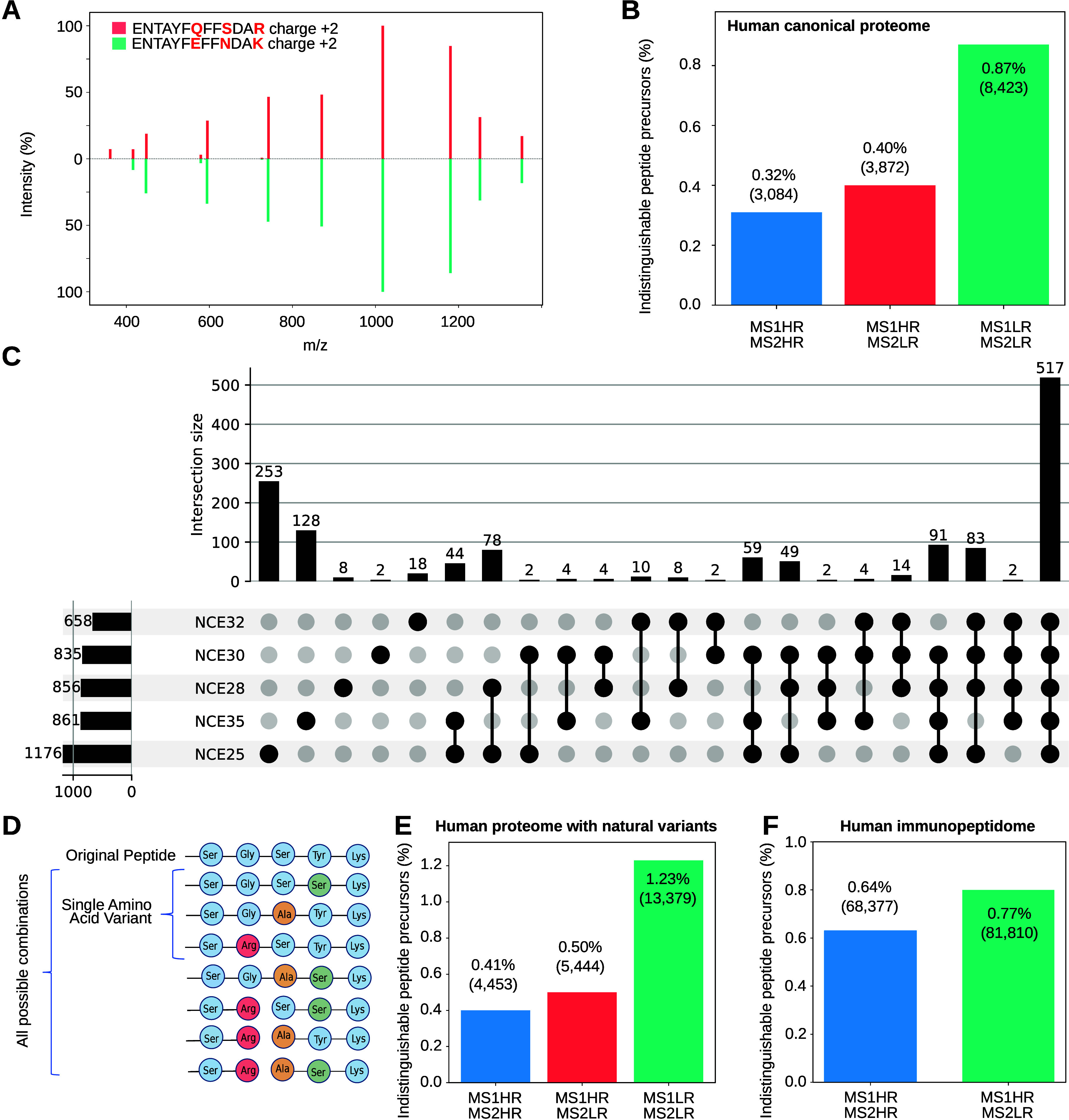
(A) Example of an indistinguishable peptide precursor pair with experimental fragmentation spectra reported in DIANN results for commercial Hela proteome extracts acquired with DIA and analyzed at 1% FDR. (B) Number and percentage of indistinguishable peptide precursors in the human canonical proteome using different mass resolution settings. (C) Number of indistinguishable peptide precursors in the human canonical proteome using different normalized collision energy values in high-resolution mass settings. (D) Schematic representation of the introduction of natural variants in the human canonical proteome. (E) Number of indistinguishable peptide precursors in the human canonical proteome with natural variants using different mass resolution settings. (F) Number of indistinguishable peptide precursors in the human immunopeptidome using different mass-resolution settings.

In this work, we performed a theoretical study to assess the prevalence of indistinguishable peptides within a given proteome using state-of-the-art retention time and spectrum prediction models. By quantifying the fraction of analytes that defy unambiguous identification, we determine the theoretical nonaccessible portion within a given proteome, and thus establish where we stand in the field.

## The Canonical Human Proteome

We started our study by assessing the prevalence of indistinguishable peptides within the canonical human proteome due to its relevance, critical importance, and widespread research interest. For this purpose, the reference UniProt database^9^ was initially submitted to in-silico tryptic digestion, allowing no miscleavages, and considering peptides with length 7–30 amino acids and charges +2 and +3. Based on these parameters, nearly a million peptide precursors (i.e., 969 900 peptide ions) were obtained. For each peptide precursor, its fragmentation spectrum and indexed retention time (iRT) were predicted with Prosit, using high-energy collision-induced dissociation (HCD) at a normalized collision energy of 28 (NCE 28).^10,11^ Then, the predicted fragmentation spectra were compared for each peptide pair within 10 ppm *m*/*z* MS1 tolerance and ± 5 iRT units (ca. 2.5 min in a 120-min chromatographic gradient), and their similarity was assessed using the normalized spectral angle function and modified cosine (10 ppm *m*/*z* MS2 tolerance).^12,13^ A normalized spectral angle threshold of 0.7 and a modified cosine threshold of 0.98 were set, indicating that peptide pairs with a similarity score above these thresholds were considered an indistinguishable pair (Figure S2).

Based on our results, we observed that the number of indistinguishable peptide precursors within the canonical human proteome represents a small percentage (0.32%, 3084 peptide precursors) in the conditions assessed. These indistinguishable peptides share a large degree of the peptide sequence as evidenced by the calculation of the edit distance, i.e., the minimum number of operations required to transform one sequence into the other (Figure S3), being 2234 peptide precursors Ile/Leu amino acid substitutions. Similar results were obtained when using another similarity score function (i.e., modified cosine; threshold 0.98; (0.34%, 3269 peptide precursors)), or when repeating the analyses with the six most intense fragment ions for each peptide (0.30%, 2906 peptide precursors), or when considering fragment ions with intensity over 5% of the most intense fragment ion (0.32%, 3147 peptide precursors) (Table S1). Accounting that each peptide sequence has two chances of being unequivocally identified, one for each charge state precursor (+2, +3), the number of peptides that are indistinguishable regardless of their charge state is reduced to 1205 peptide sequences (0.24%). Comparable percentages of indistinguishable peptide precursors and peptide sequences were achieved when generating a larger search space that accounted for charges +1 to +4 and allowed for one miscleavage (Table S2). Therefore, we conclude that, when using state-of-the-art high-resolution mass analyzers (e.g., orbital ion traps and time-of-flight) for the analysis of peptide precursors and their fragments, the theoretical nonaccessible portion for the canonical human proteome is remarkably low.

Recent studies in proteomics have revisited the use of low-resolution linear ion traps for specific proteomics applications due to their superior sensitivity and speed.^14−16^ Therefore, we wanted to further explore whether the aforementioned observations hold in scenarios in which high-resolution (HR) mass analyzers are replaced for low-resolution (LR) mass analyzers for the analysis of peptide precursors (MS1) and/or peptide fragments (MS2). The predicted spectra comparison was repeated accounting for a mass tolerance of 0.5 Da in the peptide fragments (MS1HR-MS2LR), or for both the peptide precursors and peptide fragments (MS1LR-MS2LR). Our analysis revealed that there is also a remarkably small prevalence of indistinguishable peptide precursor pairs in low-resolution settings, either MS1HR-MS2LR or MS1LR-MS2LR, with less than 1% of closely eluting tryptic peptide precursors in the canonical human proteome showing indistinguishable fragmentation relative intensities (see Figure 1B, as well as Table S3).

Finally, we were interested in assessing how the nonaccessible portion of the canonical human proteome changes with different fragmentation settings. Therefore, we explored the number and overlap of indistinguishable peptide precursors by predicting fragmentation spectra in five different collision energy settings, ranging from 25 to 35 NCE (Table S4). We observed that the population of indistinguishable peptide precursors changes across the different collision energies, and therefore adjusting the collision energy can be an effective strategy for resolving some indistinguishable peptide pairs. Only a small percentage of peptides remain indistinguishable regardless of the collision energy setting used, with the vast majority of unresolved cases involving Ile/Leu amino acid substitutions (Figure 1C). Excluding Ile/Leu amino acid substitutions, only 0.05%, i.e., 517 peptide precursors for MS1HR-MS2HR, 0.11% (1088 precursors) for MS1HR-MS2LR, and 0.48% (4643 precursors) for MS1LR-MS2LR remain indistinguishable, and when accounting for peptide precursors indistinguishable in both charge states these percentages are reduced to 0.01% (72 peptide sequence) in MS1HR-MS2HR, 0.03% (278 peptide sequence) in MS1HR-MS2LR, and 0.14% (1365 peptide sequence) in MS1LR-MS2LR. Consequently, we can conclude that the theoretical nonaccessible portion of the canonical human proteome by LC-MSMS is singnificantly low in both high- and low-resolution settings.

## The Human Proteome with Natural Variants

Building on our prior findings, we wanted to address whether we had a similar scenario when accounting for all natural variants that can occur in human populations in the form of amino acid substitutions. These natural variants often impact protein structure, function, and interactions, and their study is relevant to reveal insights into molecular mechanisms, disease susceptibility, drug response, and personalized medicine. Therefore, we broadened our analyses and evaluated the number of indistinguishable peptides in larger search spaces.

For this purpose, we first expanded the original canonical human proteome by accounting for all the single-amino acid variations (SAVs) documented in UniProt as natural variants, and accommodating all possible combinations (Figure 1D). This compilation resulted in a dataset consisting of 2 081 412 different peptide precursors (+2 and +3). Peptides with more than 20 potential variants were excluded, resulting in the removal of only 75 peptides. After introducing all amino acid variants and their combinations, a tryptic in-silico digestion was performed, followed by fragmentation spectra prediction and comparison, using the same parameters described above. The incorporation of SAVs led to a significant increase in the number of indistinguishable peptide precursors, up to 12.78% of the total number of peptide precursors in MS1LR-MS2LR, 7.69% in MS1HR-MS2LR, and ∼5.19% in MS1HR-MS2HR. When accounting for peptide precursors indistinguishable in both charge states these percentages were reduced to 3.81% (39 668 peptide sequences) of the total number of peptide sequences in MS1HR-MS2HR, 5.40% (56 250 peptide sequences) in MS1HR-MS2LR, and 9.55% (99 432 peptide sequences) in MS1LR-MS2LR (Table S5). However, the scenario of considering all possible SAV combinations in the studied peptides is probably far from reality. Indeed, upon closer examination, ∼78% of the indistinguishable peptide precursors could be traced back to the same original peptide sequences. This indicates that the observed increase in indistinguishable peptide precursors was due to the generation of many permuted instances from the same canonical sequence (Figure S4). This highlights the importance of using prior genomic information of the population or the subjects under study to reduce the search space and successfully resolve assignment ambiguities in proteogenomics studies.

To refine our analyses, we adjusted our methodology to limit the analysis to allow one SAV per peptide at a time and thus generate a more-conservative scenario. This adjustment significantly reduced the percentage of indistinguishable peptides to <2%, even in MS1LR-MS2LR resolution, aligning closely with the low uncertainty rates observed in the canonical human proteome (Figure 1E).

Our results show that when accounting for all potential modifications and their combinations, the number of indistinguishable peptide precursors increase significantly, and highlight the challenges faced in proteogenomics studies depending on the availability of prior genomic information, and the number of SAV considered within the search space.

## The Human Immunopeptidome

Finally, in our aim to evaluate the number of indistinguishable peptides in larger search spaces, we set out to explore the immunopeptidome. The immunopeptidome is a domain inherently more complex due to its magnitude, diversity, and potential sequence overlap among the presented peptides. Immunopeptidomics, particularly focusing on MHC Class I, holds great importance in the realm of cancer immunotherapy, and the identification of tumor-specific peptides presented by MHC Class I molecules is central to the development of peptide-based vaccines and adoptive T-cell therapies.^17^ To explore the nonaccessible portion of the human immunopeptidome, the reference UniProt database was used to generate in-silico all peptides in sliding windows with an offset of one amino acid, and peptide lengths ranging from 8 to 11 amino acids.^18^ However, not every peptide is presented on the cell surface by major histocompatibility complex (MHC) molecules, and its presentability depends on factors such as peptide-MHC binding affinity and protein expression, among others. We employed the state-of-the-art netMHC-pan model (version 4.1)^19^ to predict the MHC Class I binding affinity across a broad spectrum of 95 HLA-A, HLA-B, and HLA-C alleles. Over 5 million peptides were predicted as strong binding candidates across at least one allele. For each candidate peptide, its fragmentation spectrum and iRT were predicted with the Prosit HCD model for nontryptic peptides at NCE28 in charge +1 and +2.^10^ Similarly, for each peptide pair within 10 ppm *m*/*z* MS1 tolerance and ±5 iRT units (ca. 2.5 min in a 120-min chromatographic gradient), their similarity was assessed using the normalized spectral angle.

Our analyses show that despite the increased number in peptide sequences inherent to the human immunopeptidome, the percentages of indistinguishable peptide precursors remains remarkably low, with ∼0.64% (68 377 peptide precursors) in MS1HR-MS2HR, and 0.77% (71 698 peptide precursors) in MS1HR-MS2LR (see Figure 1F, as well as Table S6). The examination of low-resolution MS1 and MS2 settings was not performed in this study, due to computational limitations.

Finally, we also incorporated the natural variants described in UniProt to explore the impact of genetic variations on the immunopeptidome. We generated ∼3 million additional mutated peptides by introducing single-point mutations, of which 381 889 were predicted to exhibit high peptide-MHC binding affinities. We predicted and compared MS1 and MS2 using the same parameters described above for these peptide precursors. A slight increase in the number of indistinguishable peptide precursors was observed, being 0.68% (77 077 peptide precursors) in MS1HR-MS2HR, and 0.75% (85 374 peptide precursors) in MS1HR-MS2LR indistinguishable from other peptide precursors (Table S6). However, when assessing the number of peptide precursors indistinguishable in both charge states these percentages were reduced to 0.48% (27 062 peptide sequences) and 0.53% (29 864 peptide sequences), respectively, which highlights that the current analytical tools and methods employed are effective at differentiating peptides in a complex immunopeptidomic environment.

## Conclusion and Outlook

In this study, we have performed a theoretical assessment of indistinguishable peptides in mass-spectrometry-based proteomics within a set of given proteomes using state-of-the-art retention time and spectrum prediction models. By quantifying the fraction of analytes that challenge unambiguous identification, we have defined the theoretical nonaccessible portion of the proteome within the human canonical proteome and immunopeptidome. Despite certain design limitations—such as the choice of the similarity function, the specific deep learning prediction model employed, the exclusive use of *b* and *y* fragment ions, and the priorization of presented immunopeptides via the netMHC-pan model—we observed a low number of indistinguishable peptides within the evaluated search spaces. This finding underscores the effectiveness of current analytical approaches for proteome and immunopeptidome analyses with mass spectrometry. Not only is the number of indistinguishable peptides low, but their impact on protein identification is further mitigated by the presence of other distinguishable unique peptides within the same protein.

It is important to note that, beyond the aforementioned technical limitations, this constitutes a theoretical study aimed at identifying pairs of indistinguishable peptide precursors, which cannot capture all the details and nuances of a real proteomics experiment. In actual experimental proteomics studies, the portion of a given proteome that is identified is influenced not only by the number of distinguishable and indistinguishable peptides, but by various other factors, including charge distribution, peptide ionization efficiency, protein quantities, sample dynamic range, sample matrix, and the statistical models employed, among others.

However, the future theoretical assessment of additional more complex proteomes remains of high interest, particularly the ones that include the analysis of post-translational modifications like phosphorylated peptides, for which reliable fragmentation prediction models are not yet generally available. Other scenarios include the analysis of the proteome of multiple species (*metaproteomics*), or even the entire proteome universe defined as all the protein entries in Uniprot, which would benefit from the introduction of additional analytical dimensions, e.g., ion mobility, and the use of highly efficient algorithmic strategies.

## Data Availability

The code used for the analyses presented in this study is openly available. It can be accessed and downloaded from the following GitHub repository: https://github.com/proteomicsunitcrg/MSCI. Users are encouraged to refer to the repository for the most up-to-date package version, documentation, associated data, or examples.

## Supporting Information Available

Materials and Methods section; **Figure S1:** Examples of indistinguishable peptide precursor pairs; **Figure S2:** Distribution of similarity scores for peptide pairs with Ile/Leu substitutions and randomly selected peptide pairs; **Figure S3:** Distribution of “Edit Distances” among all peptide pairs and indistinguishable peptide pairs; **Figure S4:** Distribution of indistinguishable peptide pairs with natural variants by origin; **Table S1:** Number of indistinguishable peptide precursors in the human canonical proteome; **Table S2:** Number of indistinguishable peptide precursors and peptide sequences in the human canonical proteome accounting extended charges and miscleavages; **Table S3:** Number of indistinguishable peptide precursors in the human canonical proteome using different spectra filtering methods; **Table S4:** Number of indistinguishable peptide precursors in the human canonical proteome using different values of normalized collision energy; **Table S5:** Number of indistinguishable peptide precursors and peptide sequences in the human canonical proteome accounting for known natural variants; **Table S6:** Number of indistinguishable peptides and peptide precursors in the human immunopeptidome; **Table S7:** Number of all the peptide combinations compared at high- and low-resolution MS1 for the different assessed scenarios (PDF)
