# Supplementary material for: Theoretical Assessment of Indistinguishable Peptides in Mass Spectrometry-Based Proteomics
Source: Anal Chem. 2024 Sep 25;96(40):15829–33. doi: 10.1021/acs.analchem.4c02803 (PMC11465223; doi:10.1021/acs.analchem.4c02803)
Supplement: Supplementary file 1 — ac4c02803_si_001.pdf [file ac4c02803_si_001.pdf]

Supporting Information

Letter

Theoretical assessment of indistinguishable peptides in mass spectrometry-based proteomics

Zahra Elhamraoui[1,2], Eva Borràs[1,2], Mathias Wilhelm[3,4], Eduard Sabidó[1,2,*]

1. Centre for Genomic Regulation, The Barcelona Institute of Science and Technology (BIST), Dr. Aiguader 88, Barcelona, 08003, Spain.

2. Univeristat Pompeu Fabra, Dr. Aiguader 88, Barcelona, 08003, Spain.

3. Computational Mass Spectrometry, Technical University of Munich, Freising, Germany.

4. Munich Data Science Institute (MDSI), Technical University of Munich, Garching, Germany.

Table of Contents

- Materials and Methods
- Supplementary Figure 1
- Supplementary Figure 2
- Supplementary Figure 3
- Supplementary Figure 4
- Supplementary Table 1
- Supplementary Table 2
- Supplementary Table 3
- Supplementary Table 4
- Supplementary Table 5
- Supplementary Table 6
- Supplementary Table 7

Materials and Methods

Theoretical prediction of spectra

A reference UniProt database containing all reviewed human protein entries was used (accessed September 2023) for spectra prediction of the human proteome. An *in-silico* tryptic digestion was performed by applying digestion rules that cleave at the carboxyl side of lysine (K) and arginine (R), except when either is followed by a proline (P). For the analyses of the canonical proteome with and without natural variants two strategies were followed. The first strategy was a peptide digestion allowing no miscleavages, and considering peptides with length 7-30 amino acids and charges +2 and +3. The second strategy, more comprehensive, allowed for one miscleavage, and considered peptides with length 7-30 amino acids, and charges +1, +2, +3 and +4. In both cases cysteines were considered as carbamidomethylated. For each peptide precursor obtained, its fragmentation spectrum and indexed retention time (iRT) were predicted with Prosit using the high-energy collision-induced dissociation model (Prosit_2020_intensity_HCD) at normalised collision energies (NCE) of 25, 28, 30, 32 and 35. All predicted libraries are available at https://doi.org/10.5281/zenodo.12748934.

For the analyses of the immunopeptidome, the aforementioned reference UniProt human database was used to generate *in-silico* all peptides in sliding windows with an offset of one amino acid, and peptide lengths ranging from 8 to 11 amino acids. As the Prosit model was not trained on peptides containing free cysteine side chains, cysteine-containing peptides were removed from the subsequent analyses. The list of potential immunopeptides was reduced using the netMHC-pan model (version 4.1) that predicted the MHC Class I binding affinity across a broad spectrum of 95 HLA-A, -B, and -C alleles. Over 5 million peptides were predicted as strong binding candidates across at least one allele. For each candidate peptide, its fragmentation spectrum and iRT were predicted with the Prosit HCD model for non-tryptic peptides at NCE28 in charge +1 and +2 (Prosit_2020_intensity_HCD). All predicted libraries are available at https://doi.org/10.5281/zenodo.12748934.

The predicted fragmentation spectra were compared for each peptide pair within 10 ppm (high-resolution) or 0.5 Da (low-resolution) m/z MS1 tolerance and +/- 5 iRT units (*ca.* 2.5 min in a 120-min chromatographic gradient). Spectral similarity was assessed using the normalised spectral angle function and modified cosine. The normalized spectral angle was calculated with the formula $\lambda = 1-((2\cdot\cos^{-1}(S_1\cdot S_2))/\pi)$ and *matchms* (version 0.18.0) was used for the calculation of the modified cosine (0.001 Da m/z MS2 tolerance).

A peptide pair was considered indistinguishable when having a normalized spectral angle threshold of 0.7. This threshold was selected to cover most (>95%) of the experimental variability within spectra acquired for the exact same peptide,[1] and account for the inherent prediction error of the fragmentation model (Prosit),[2] while maintaining the ability to discern between distinguishable and indistinguishable peptide pairs. The bona fide of the selected threshold was verified based on the classification of randomly selected peptide pairs (mostly distinct) compared with those with one Ile/Leu substitution. Although some Ile/Leu substitutions might slightly alter the peptide fragmentation pattern,[3] these Ile/Leu-substitution peptide pairs were considered as positive controls for

indistinguishable peptides. The selected threshold classified Ile/Leu-substitution peptides as indistinguishable while classifying the vast majority of randomly selected peptide pairs as distinguishable (Supplementary Figure 2) . Moreover, expert manual inspection of the spectra from other indistinguishable peptide pairs was also a mean of verification of the selected threshold. The same rationale was used to establish the modified cosine threshold to 0.98.

Experimental results related to Figure 1A and Supplementary Figure 1

Commercial HeLa tryptic digests (Pierce HeLa Protein Digest Standard, Thermo Scientific #1862824) were analyzed in an LTQ-Orbitrap Eclipse mass spectrometer (Thermo Fisher Scientific) coupled to an EASY-nLC 1200 (Thermo Fisher Scientific). Peptides were loaded directly onto the analytical column and were separated by reversed-phase chromatography using a 50-cm column with an inner diameter of 75 μm, packed with 2 μm C18 particles spectrometer (PepMap RSLC C18, Thermo Scientific #ES803A). Chromatographic gradients started at 95% buffer A and 5% buffer B with a flow rate of 300 nl/min and gradually increased to 25%buffer B and 75% A in 105 min and then to 40% buffer B and 60% A in 15 min. After each analysis, the column was washed for 10 min with 1000% buffer B. Buffer A: 0.1% formic acid in water. Buffer B: 0.1% formic acid in 80% acetonitrile.

The mass spectrometer was operated in positive ionization mode with an EASY-Spray nanosource with spray voltage set at 1.4 kV and source temperature at 305 °C. The instrument was operated in data-independent acquisition mode, with a full MS scans over a mass range of m/z 500-900 in the orbitrap (30K resolution) and 64 ms maximum injection time with auto gain control (AGC) set to 1E6. In each cycle of data-independent acquisition analysis, following each full MS scan, a set of 40 consecutive windows of 10 Da each over a mass range of 500-900 m/z. MS2 scan range was set from 350 to 1850 m/z, with an AGC target of 1E6 and a maximum injection time of 54 ms. Depending on the run, a normalized collision energy of either 25, 28, 30, 32 and 35 was used for higher-energy collisional dissociation (HCD) fragmentation. Fragment ion spectra were acquired in the orbitrap mass analyzer at 30,000 resolution. All data were acquired with Xcalibur software.

DIA raw files were processed with DIANN v1.8.1 using predicted libraries from the Swiss-Prot human database (reviewed entries, accessed September 2023). Oxidation of methionine and N-terminal protein acetylation were set as variable modifications whereas carbamidomethylation on cysteines was set as a fixed modification, trypsin was the chosen enzyme, and up to two missed cleavages were allowed. For identification, precursor and protein q-value cut off was set to 0.01 and quantification data filtering was set to “Q-value” in which only individual observations above the q-value threshold were considered.

The mass spectrometry proteomics data have been deposited at the ProteomeXChange Consortium via the PRIDE repository with identifier PXD000000.

Bibliography

1. Toprak UH, Gillet LC, Maiolica A, Navarro P, Leitner A, Aebersold R. Conserved peptide fragmentation as a benchmarking tool for mass spectrometers and a discriminating feature for targeted proteomics. Mol Cell Proteomics. 2014 Aug;13(8):2056-71. doi: 10.1074/mcp.O113.036475

2. Gessulat S, Schmidt T, Zolg DP, Samaras P, Schnatbaum K, Zerweck J, Knaute T, Rechenberger J, Delanghe B, Huhmer A, Reimer U, Ehrlich HC, Aiche S, Kuster B, Wilhelm M. Prosit: proteome-wide prediction of peptide tandem mass spectra by deep learning. Nat Methods. 2019 Jun;16(6):509-518. doi: 10.1038/s41592-019-0426-7

3. Wu HT, Riggs DL, Lyon YA, Julian RR. Statistical Framework for Identifying Differences in Similar Mass Spectra: Expanding Possibilities for Isomer Identification. Anal Chem. 2023 May 2;95(17):6996-7005. doi: 10.1021/acs.analchem.3c00495.

Supplementary Figure 1: Examples of experimental fragmentation spectra from indistinguishable peptide precursor pairs reported in DIANN results for HeLa protein extracts acquired in DIA at 1% FDR; A) KYGVTTIVR and KYGVTTLVR representing Ile/Leu permutation; B) ISMPDFDLNLK and ISMPDIDFNLK with amino acid permutations, C) IKGDVDVSAPK and IKGDVDVTGPK representing different amino acids sequences with the same mass/charge and overlapping retention times.

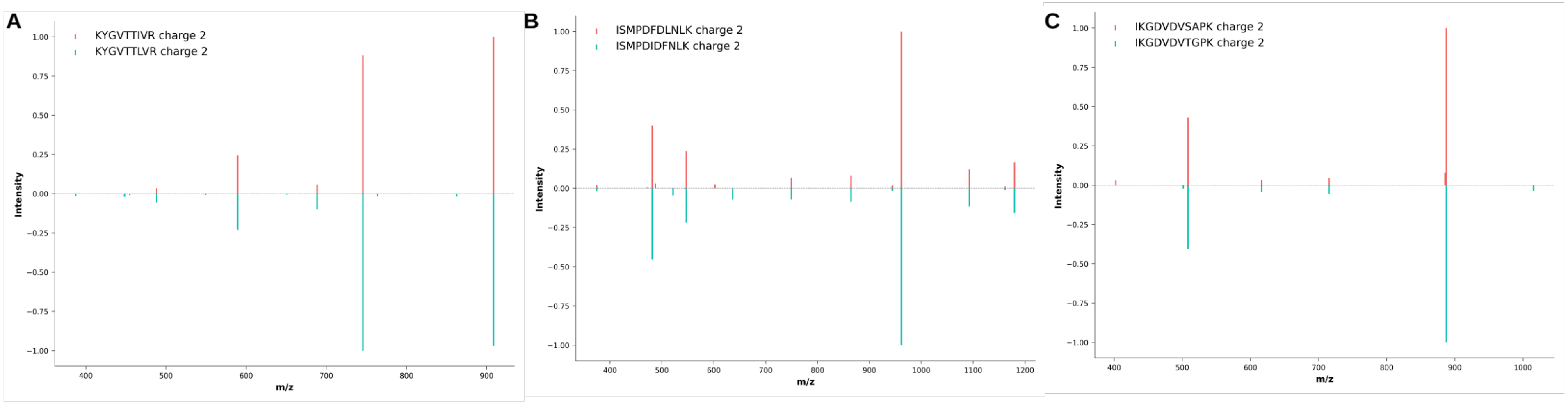

Supplementary Figure 2: Distribution of similarity scores based on the normalized spectral angle (A), and modified cosine (B) for peptide pairs with Ile/Leu substitutions (n=1,142) and randomly selected peptide pairs (n=1,142; n=11,420; and n=114,200). The selected threshold for indistinguishable peptides is marked with a red line. Peptide pairs were obtained from the canonical proteome using high mass resolution (HR) in MS1 and MS2, charges +2/+3, no miscleavages, and with a 5% intensity filter.

A

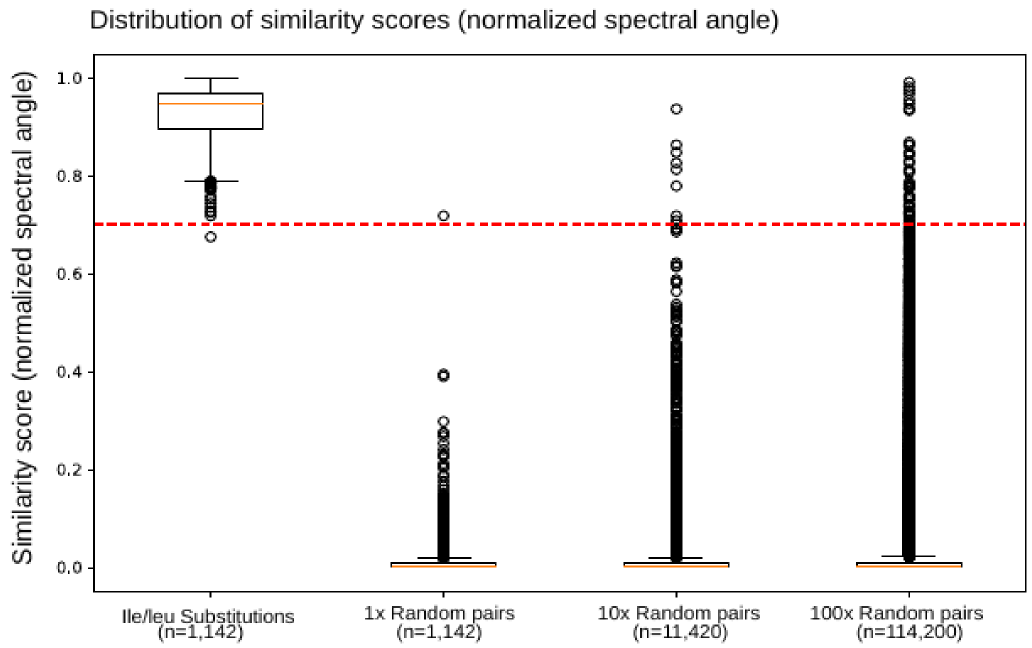

B

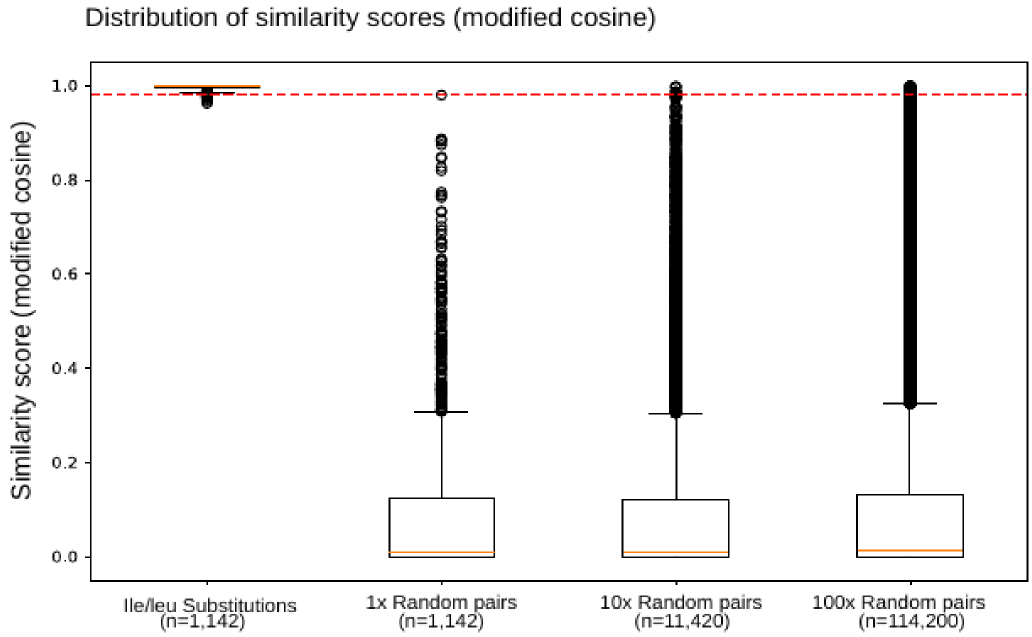

Supplementary Figure 3: Distribution of Edit Distances calculated with the Levenshtein Distance among all peptide pairs and among the indistinguishable peptide pairs obtained in the evaluation of the canonical proteome using high mass resolution (HR) in MS1 and MS2.

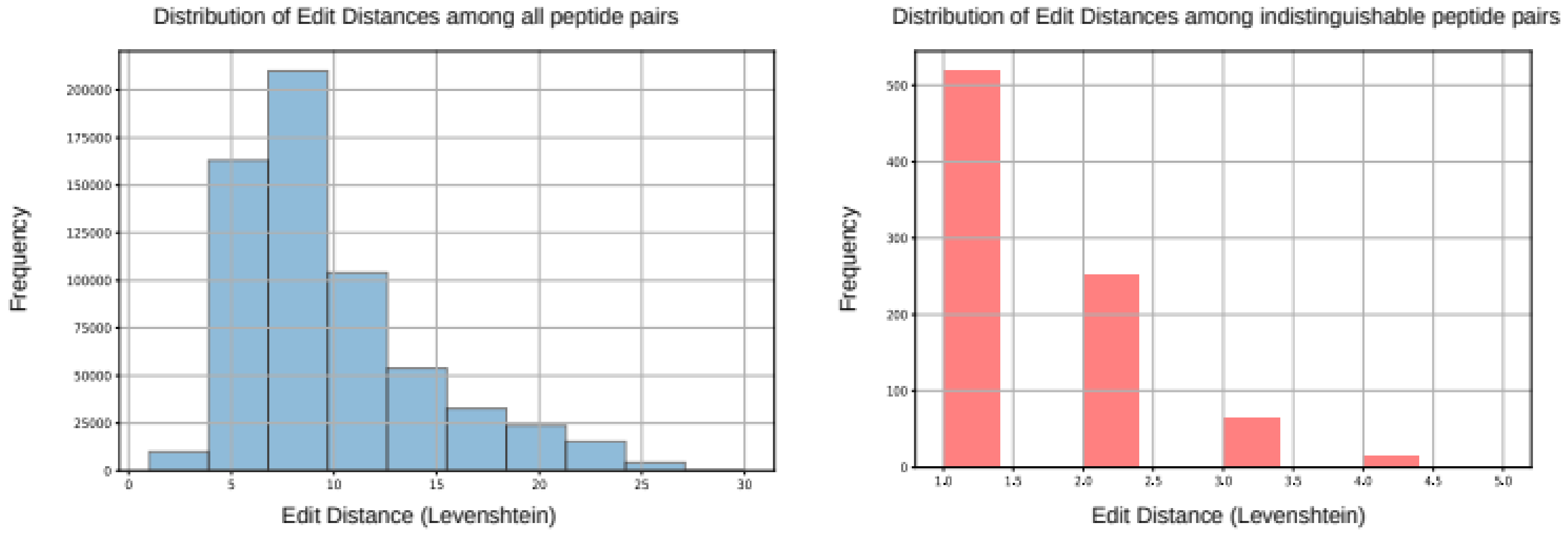

Supplementary Figure 4: Distribution of indistinguishable peptide pairs by origin when evaluating the canonical proteome with all potential combination of natural variants using high mass resolution (HR) in MS1 and MS2.

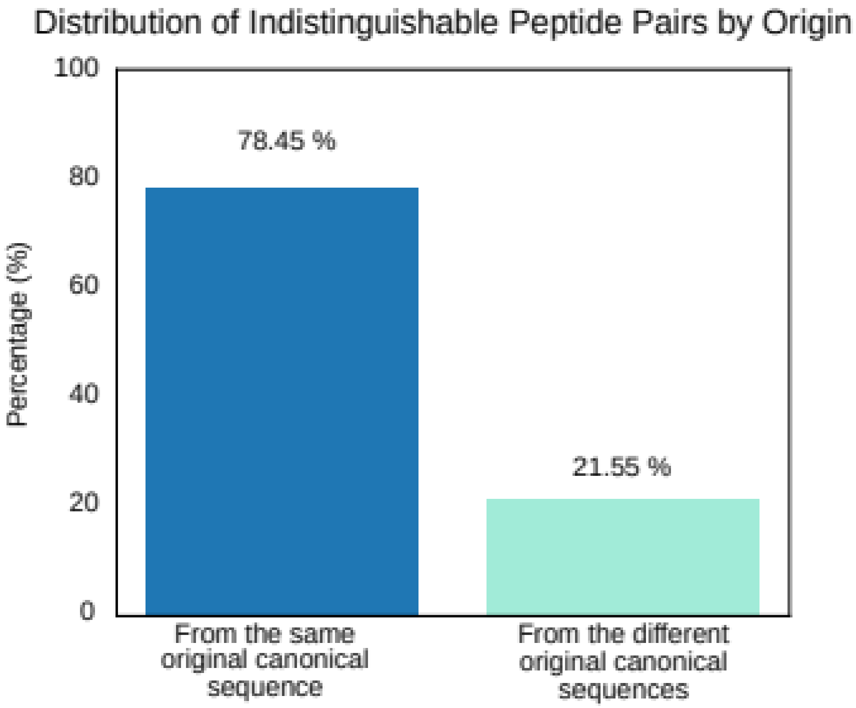

Supplementary Table 1: Number of indistinguishable peptide precursors in the human canonical proteome using high mass resolution (HR) in MS1 and MS2, two different similarity score functions, and three different spectra filtering methods.

**Resolution**	**Similarity score fucntion**	**Spectra filtering method**	**Number of indistinguishable peptide precursors (%)**
MS1HR-MS2HR	Normalised Spectral angle	Top6 Peaks	2,906 (0.30%)
		Only peaks above 5%	3,147 (0.32%)
		Whole spectra	3,084 (0.32%)
	Cosine	Top6 Peaks	3,482 (0.36%)
		Only peaks above 5%	3,659 (0.38%)
		Whole spectra	3,269 (0.34%)

Supplementary Table 2: Number of indistinguishable peptide precursors and peptide sequences in the human canonical proteome accounting for charges +1, +2, +3, and +4, and allowing one miscleavage.

		**Number of indistinguishable peptide precursors**		**Number of indistinguishable peptides sequences**	
	**Resolution**	**All**	**Excluding Ile/Leu substitutions**	**All**	**Excluding Ile/Leu substitutions**
Human canonical proteome (+1/+2/+3/+4, one miscleavage)<br><br>*Total: 4,848,780 peptide precursors*<br>*Total: 1,212,195peptide sequences*	MS1HR-MS2HR	14,540 (0.30%)	4,500 (0.09%)	2,514 (0.21%)	70 (0.01%)
	MS1HR-MS2LR	16,195 (0.33%)	6,210 (0.13%)	2,596 (0.21%)	161 (0.01%)
	MS1LR-MS2LR	33,126 (0.68%)	23,343 (0.48%)	2,686 (0.22%)	256 (0.02%)

Supplementary Table 3: Number of indistinguishable peptide precursors in the human canonical proteome using high (HR) and low (LR) mass resolution and three different spectra filtering methods.

Resolution	Method	Number of indistinguishable peptide precursors (%)
MS1HR-MS2LR	Top6 Peaks	3,669 (0.38%)
	Only peaks above 5%	3,993 (0.41%)
	Whole spectra	3,872 (0.40%)
MS1LR-MS2LR	Top6 Peaks	8,390 (0.87%)
	Only peaks above 5%	8,977 (0.93%)
	Whole spectra	8,423 (0.87%)

Supplementary Table 4: Number of indistinguishable peptide precursors in the human canonical proteome using different values of normalized collision energy in high mass resolution.

Resolution	NCE	Number of indistinguishable peptide precursors and percentage
MS1HR-MS2HR	25	3,397 (0.35%)
	28	3,084 (0.32%)
	30	3,062 (0.31%)
	32	2,884(0.30%)
	35	3,087 (0.32%)

Supplementary Table 5: Number of indistinguishable peptide precursors and peptide sequences in the human canonical proteome accounting for known natural variants.

		Number of indistinguishable peptide precursors		Number of indistinguishable peptides sequences	
	**Resolution**	**All**	**Excluding Ile/Leu substitutions**	**All**	**Excluding Ile/Leu substitutions**
Natural variants with all possible combinations<br><br>*Total: 2,081,412 peptide precursors*<br>*Total: 1,040,706 peptide sequences*	MS1HR-MS2HR	108,031 (5.19%)	62,904 (3.02%)	39,668 (3.81%)	11,891 (0.57%)
	MS1HR-MS2LR	159,998 (7.69%)	105,380 (5.06%)	56,250 (5.40%)	27,555 (2.64%)
	MS1LR-MS2LR	266,097 (12.78%)	213,647 (10.26%)	99,432 (9.55%)	71,735 (6.89%)
Natural variants with one single-amino acid substitution at once<br><br>*Total: 1,091,120 peptide precursors*<br>*Total: 545,560 peptide sequences*	MS1HR-MS2HR	4,453 (0.41%)	1,233 (0.11%)	1,786 (0.33%)	174 (0.03%)
	MS1HR-MS2LR	5,444 (0.50%)	2,308 (0.21%)	2064 (0.37%)	503 (0.09%)
	MS1LR-MS2LR	13,379 (1.23%)	10,301 (0.94%)	4,492 (0.82%)	2,955 (0.54%)
Reference human canonical proteome without natural variants<br><br>*Total: 969,900 peptide precursors*<br>*Total: 484,950 peptide sequences*	MS1HR-MS2HR	3,084 (0.32%)	856 (0.09%)	1,205 (0.24%)	89 (0.01%)
	MS1HR-MS2LR	3,872 (0.40%)	1,727 (0.18%)	1,435 (0.29%)	366 (0.07%)
	MS1LR-MS2LR	8,423 (0.87%)	6,318 (0.65%)	2,576 (0.53%)	1,524 (0.31%)

Supplementary Table 6: Number of indistinguishable peptides and peptide precursors in the human immunopeptidome

		**Number of indistinguishable peptide precursors**		**Number of indistinguishable peptides sequences**	
	**Resolution**	**All**	**Excluding Ile/Leu substitutions**	**All**	**Excluding Ile/Leu substitutions**
Human immunopeptidome<br><br>*Total: 10,607,028 peptide precursors*<br>*Total: 5,303,514 peptide sequences*	MS1HR-MS2HR	68,377 (0.64%)	29,980 (0.28%)	21,496 (0.41%)	3,529 (0.07%)
	MS1HR-MS2LR	71,698 (0.77%)	33,346 (0.31%)	22,403 (0.42%)	4,452 (0.08%)
Human immunopeptidome with one single-amino acid substitution at once<br><br>*Total: 11,370,806 peptide precursors*<br>*Total: 5,685,403 peptide sequences*	MS1HR-MS2HR	77,077 (0.68%)	31,537 (0.28%)	27,062 (0.48%)	4,151 (0.07%)
	MS1HR-MS2LR	85,374 (0.75%)	39,945 (0.35%)	29,864 (0.53%)	7,000 (0.12%)

Supplementary Table 7: Number of all the peptide combinations compared at high- (HR) and low-resolution (LR) MS1 for the different assessed scenarios.

**Dataset**	**MS1HR pairs**	**MS1LR pairs**
Human canonical proteome (+2/+3, no miscleavage)	1,103,925	39,677,045
Human canonical proteome (+1/+2/+3/+4, one miscleavage)	10,550,829	534,860,821
Human canonical proteome accounting for natural variants with all possible combinations	6,166,158	157,891,235
Human canonical proteome accounting for natural variants with one single-amino acid substitution at once	1,326,319	48,537,151
Human immunopeptidome	216,384,689	-
Human immunopeptidome accounting for natural variants with one single-amino acid substitution at once	237,040,053	-
